# Recreational Exercising and Self-Reported Cardiometabolic Diseases in German People Living with HIV: A Cross-Sectional Study

**DOI:** 10.3390/ijerph182111579

**Published:** 2021-11-04

**Authors:** Camilo Germán Alberto Pérez-Chaparro, Felipe B. Schuch, Philipp Zech, Maria Kangas, Michael A. Rapp, Andreas Heissel

**Affiliations:** 1 Outpatient Clinic—Centre for Sports Medicine, Department of Sports & Health Sciences, University of Potsdam, 14469 Potsdam, Germany; 2 Department of Sports Methods and Techniques, Federal University of Santa Maria, Santa Maria 97105, Brazil; felipe.schuch@ufsm.br; 3 Social and Preventive Medicine, Department of Exercise and Health Sciences, University of Potsdam, 14469 Potsdam, Germany; phzech@uni-potsdam.de; 4 Centre for Emotional Health, Department of Psychology, Macquarie University, Sydney 2109, Australia; maria.kangas@mq.edu.au; 5 Social and Preventive Medicine, Department of Sports and Health Sciences, Intra-Faculty Unit “Cognitive Sciences”, Faculty of Human Science, and Faculty of Health Sciences Brandenburg, Research Area Services Research and e-Health, University of Potsdam, 14469 Potsdam, Germany; michael.rapp@uni-potsdam.de (M.A.R.); andreas.heissel@uni-potsdam.de (A.H.)

**Keywords:** HIV, exercise, cardiovascular diseases, metabolic disease, sedentary

## Abstract

Exercise is known for its beneficial effects on preventing cardiometabolic diseases (CMDs) in the general population. People living with the human immunodeficiency virus (PLWH) are prone to sedentarism, thus raising their already elevated risk of developing CMDs in comparison to individuals without HIV. The aim of this cross-sectional study was to determine if exercise is associated with reduced risk of self-reported CMDs in a German HIV-positive sample (*n* = 446). Participants completed a self-report survey to assess exercise levels, date of HIV diagnosis, CD4 cell count, antiretroviral therapy, and CMDs. Participants were classified into exercising or sedentary conditions. Generalized linear models with Poisson regression were conducted to assess the prevalence ratio (PR) of PLWH reporting a CMD. Exercising PLWH were less likely to report a heart arrhythmia for every increase in exercise duration (PR: 0.20: 95% CI: 0.10–0.62, *p* < 0.01) and diabetes mellitus for every increase in exercise session per week (PR: 0.40: 95% CI: 0.10–1, *p* < 0.01). Exercise frequency and duration are associated with a decreased risk of reporting arrhythmia and diabetes mellitus in PLWH. Further studies are needed to elucidate the mechanisms underlying exercise as a protective factor for CMDs in PLWH.

## 1. Introduction

Life expectancy of younger people diagnosed with HIV is approximately 54 years [1] This reduced life expectancy can be attributed to the increased prevalence of CMDs as described in European PLWH (3.7–5%) [2], including German PLWH compared to individuals without HIV (12.8% vs. 10.4%, *p* < 0.01), with premature onset and higher prevalence of CMDs in PLWH older than 40 years [3].

The incidence of CMDs can be explained by traditional risk factors (e.g., age, high waist-to-hip ratio, symptomatic HIV) [4], and the role of ART and HIV chronic inflammation [5,6]. In addition, health behaviors like smoking [7] and/or physical inactivity [8] are highly prevalent in people with HIV [9].

Conversely, exercise is beneficial for preventing CMDs in the general population [10]. Specifically, cross-sectional studies have found that exercise has a protective factor against hypertension (odds ratio (OR) = 0.49, 95% CI: 0.29–0.84 to OR = 0.92, 95% CI: 0.87–0.98) and diabetes (OR = 0.46, 96% CI: 0.25-0.86 to OR = 0.87, 95% CI: 0.80–0.95) [11,12], and people performing exercise ≥ 150 min per week are 42% less likely to have a cardiovascular disease [13].

Recent meta-analyses indicate that exercise has a beneficial effect on PLWHs’ physical health (including increased aerobic [14] and strength [15] capacity), favoring a better cardiorespiratory fitness (CRF) [16]. In contrast, physical inactivity is associated with the development of hypertension (prevalence ratio (PR) = 1.46, 95% CI: 1.03–2.06, *p* = 0.03) and diabetes mellitus (PR = 1.71, 95% CI: 1.12–2.62, *p* = 0.01) in PLWH [17].

Exercise is recommended to prevent or treat CMDs in the general population. Data on the protective effect of exercise intensity, frequency, and duration on CMDs in German PLWH is scarce. The aim of this study was to investigate the role exercise has in reported CMDs in German PLWH. The objectives of this study were to (1) assess the differences in comorbidities between two groups of PLWH (sedentary = Sed and exercising = Exe) and (2) calculate the PR of reporting a CMD among the two groups in relation to self-reported: anthropometric characteristics (age, weight, BMI); number of years living with HIV; exercise characteristics (intensity, frequency, duration); and exercise status (Sed vs. Exe).

## 2. Materials and Methods

This cross-sectional study was based on data from the HIBES study [18]. Individuals were recruited from various German institutions involved in HIV/AIDS to minimize the recruitment bias. Participants were recruited from (1) the official AIDS-offices (Germany-wide), (2) the Academy Waldschlösschen e.V., (3) medical care facilities in Berlin and Germany who specialize in HIV and AIDS, (4) the consortium of HIV and AIDS specialized physicians (DAGNÄ), and (5) the Competence Network HIV/AIDS. The study was conducted from October 2010 to December 2012 over a 26-month period in both rural and urban areas of Germany. (Questionnaires (Supplementary Questionnaire S1)) were freely available for individuals to complete either in hardcopy or online format. Inclusion criteria were as follows: ≥18 years of age, diagnosed with HIV, and completed the exercise questionnaire. The survey assessed self-reported HIV and ART characteristics (years with HIV, CD4 cell count, and current ART: Truvada, Trizivir, Kivexa, Combivir, Atripia, or no combination), and the self-reported presence of a CMD (comparable to the GEDA 2014/2015-EHIS questionnaire [19]). Specifically, participants were asked to indicate yes or no if they had a CMD in addition to HIV; if the answer was affirmative, participants were asked to mark a box if they had one or more of the following CMDs: atherosclerosis, arterial hypertension, coronary heart disease (CHD), diabetes mellitus (DM), dyslipidemia, heart arrhythmia, heart insufficiency, myocardial infarction (MI), peripheral arterial disease (PAD), and stroke, before or after the HIV diagnosis. Participants were also asked to report any medication they were taking in relation to the CMD. To ensure the diagnosis was made by a medical doctor, only CMDs with the correct pharmacology treatment were considered to be valid. The survey investigated regular exercise defined as “planned, structured and repetitive bodily movement done to improve or maintain one or more components of physical fitness” [20] performed at least once a week [21] for more than one year. If the answer was affirmative, participants were asked to report the exercise type, time period, frequency, and duration. The length of exercise practice was measured in years. Exercise frequency was measured in number of sessions per week (sessions·week ^1^). Each reported type of sport was converted into metabolic equivalent of task (METs) to measure exercise intensity according to Ainsworth et al. [22] and expressed as MET·min·day^−1^ [23]. The duration of each exercise session was measured in hours per week (h·week^−1^). Details concerning intensity, duration, and frequency assessments can be found in [18]. Exercise frequency, intensity, and duration were divided in tertiles. Participants were categorized into two groups: (1) Exe: participants who performed exercise more than 12 months before completing the survey and (2) Sedentary: participants who never performed exercise, before completing the survey.

The immunological parameters of cluster differentiation four (CD4) white blood cell count were a self-report question (participants were asked to check a box with different CD4 values: <200, 200–499, and >500 cell/µL, according to the Center for Disease Control (CDC) [24]. Years living with HIV was calculated based on the year of HIV diagnosis and the time they answered the survey. Body mass index (BMI) was calculated from self-reported weight and height after the HIV diagnosis. Participants who did not answer the questions about exercise frequency, length of time, or type were excluded from this study.

### Statistical Analysis

A prior descriptive analysis including the Kolmogorov–Smirnov and Shapiro–Wilk tests, indicated a non-normal distribution of the variables. The Mann–Whitney U test was performed to evaluate any differences in age (years), height (cm), weight (kg), body mass index (BMI = kg·m^2−1^), years living with HIV (years), exercise frequency (days per week^−1^), exercise intensity (MET·min·day^−1^), and exercise duration (h per week^−1^) between the groups. The Chi-square test with a post-hoc analysis applying the Bonferroni correction [25] was used to evaluate the differences between the groups in terms of gender, CD4 cell count category according to the CDC [24], exercise frequency, intensity, and duration tertiles, and prevalence of CMDs before and after the HIV diagnosis. Fisher’s exact test was chosen in cases where one or more frequencies were less than five. A Poisson regression with robust variance was performed to calculate the PR and 95% confidence intervals [26,27] of CMDs. The relationship between CMDs after the HIV diagnosis and the different variables was analyzed as follows: the relationship to anthropometric characteristics (age, weight, and BMI) adjusted for years living with HIV in model 1; to number of years living with HIV, adjusted for age, weight and BMI in model 2; to exercise characteristics (intensity, frequency, duration) adjusted for age, weight, and BMI and diabetes mellitus in model 3; and to the current exercise status (exercising vs. sedentary) adjusted for age, weight, and BMI and diabetes mellitus in model 4. The significance level of alpha was set to <0.05. Data are presented as median and interquartile range (IQR, 1st quartile–3rd quartile) unless otherwise indicated. All statistical analyses were performed using the statistical package SPSS version 26 (IBM Corp., Armonk, NY, USA).

## 3. Results

The Exe group was significantly younger, and the proportion of females was lower compared to the Sed group. The Sed group had a greater proportion of participants with a CD4 cell count < 200 cell/µL, whereas a greater proportion of PLWH in the Exe had a CD4 cell count > 500 cell/µL. Characteristics of the cohort are shown in Table 1.

### 3.1. Exercise in PLWH

The proportion of PLWH who reported not being engaged in exercise was 37.2%. The greater proportion of exercising PLHW did so with frequency between 2 and 3 days·week^−1^, with an intensity of ≤103 MET·min·day^−1^, and total duration greater than 4 h·week^−1^, as summarized in Table 2.

### 3.2. CVD in PLWH

Overall, 8.5% of participants reported one or more CMD after the HIV diagnosis. Of these 38 participants, 79% (n = 30) reported one CMD, 16% (n = 6) reported two CMDs, and 5% (n = 2) reported three CMDs. The Exe group reported a non-significant higher proportion of CMDs (65.8%) compared to the Sed group (34.2%). No differences were found between groups for the reported prevalence of any specific CMD before or following the HIV diagnosis, as reported in Table 3.

### 3.3. Risk of Reporting a CVD after the HIV Diagnosis

For every increase in years living with HIV, PLWH were more likely to report arterial hypertension, atherosclerosis, CHD, and dyslipidemia. PLWH were less likely to report arrhythmias for every increase in hours of exercise and diabetes mellitus for every increase in exercise session per week. No relation between the reported type of ART treatment and reported CMD was found (see Table 4).

## 4. Discussion

More than the half of the participants (62.8%) in this study reported engaging in exercise at the time of assessment. These results are comparable with other studies in this field, including a Swiss HIV cohort [8], where the prevalence of physical inactivity was 44%. Furthermore, 38% of the exercise group in the current sample reported spending 2–3 days·week^−1^ engaging in exercise. These results are compatible with Schäfer et al.’s study [8] whereby 14% of their sample exercised 3–4 times per week.

The incidence of CMDs in the current sample could be explained by the HIV-related side effects and ART treatment linked to dyslipidemia, glycemic alterations, and a chronic inflammatory state [5,28,29]. In this context, the CHD and MI prevalence in this study population is not surprising as PLWH have a Framingham CHD risk score >20% [30]. Furthermore, the prevalence of hypertension in this study (9.6%) is consistent with the prevalence rate of 7.9% reported in the Silveira et al. [17] Brazilian sample of PLWH.

A noteworthy outcome from this study is that PLWH had a decreased likelihood of reporting an arrhythmia for every increase in hours of exercise per week. This is of importance since PLWH are predisposed to sudden cardiac death in part due to more arrhythmias (such as prolonged QTc intervals: 1.6–4 times more in PLWH compared to non-HIV) [31]. The reason for the increased arrhythmias arises from medications used to treat opportunistic infection in PLWH capable of prolonging QTc [31]. Another source of arrhythmias is an electrolyte disturbance in cases of gastrointestinal opportunistic infections. There is also evidence that the HIV virus has a primary relation with arrhythmias as Torsades de Pointes in the context of QTc prolongation has been described in PLWH, even in the absence of drugs or electrolyte alterations [31]. Additionally, PLWH taking ART have a lower heart rate variability (HRV) compared to the general population that could increase the risk of arrhythmias [32]. Exercise increases HRV (is associated with a better autonomic balance) in PLHW [33], by improving vagal tone to the heart and at the same time regulating sympathetic tone, that prevents against malignant arrhythmias [34]. Nevertheless, it is important to stress that high aerobic fitness in older adults could have a pro-arrhythmic effect [35].

We also found that PLWH had a decreased likelihood of reporting diabetes mellitus for every increase in exercise session per week, explained by the effects of exercise in stimulating GLUT4 (inhibited by HIV Nef protein), decreasing insulin resistance, and increasing β-cell insulin secretion (impaired in PLWH) [10,36].

## 5. Limitations

Some limitations need to be considered. First, although a notable strength was the large sample size (n = 516 PLWH), the sample might not be nationally representative given the sample was predominantly male, with a distribution representative for PLWH in more economically developed countries [37] and taking into consideration that European women with HIV are less likely to have CMD [3]. Second, the assessment for HIV and CMDs prevalence and risk factors were based on self-report measures, including retrospective evaluations, whilst access to participants’ medical records was not available; sole reliance on self-report may therefore have increased the risk of recall and social bias. Third, exercise was not objectively assessed through accelerometry or a standard validated physical activity questionnaire, such as the International Physical Activity Questionnaire (IPAQ). Rather, exercise was assessed using self-administered questions regarding exercise taking place in participants’ leisure time without considering the exercise carried out during worktime. Fourth the statistical models were not adjusted by participating institutions due to the nature of anonymous assessment, and no mathematical correction or adjustment was made for multiple comparisons, acknowledging that for the risk of reporting a CVD after the HIV diagnosis—as summarized in Table 4—the statistically significant findings were at *p* < 0.01. Furthermore, due to the cross-sectional nature of the study, it is not possible to determine causality between variables found to be significantly associated with each other. Finally, due to the cross-sectional design, the CMDs incidence could not be determined, thus, limiting understanding of the comorbidity trends that have emerged.

## 6. Implications for Research

Exercise is prescribed in cases of CMDs for its health benefits [10,38]. The role of exercise as a protective factor against CMDs in comparison to physical inactivity in PLWH is of great value in the treatment of CMDs. Due to the cross-sectional study design, a comprehensive evaluation between exercise and inactivity and their corresponding associations with CMDs in PLWH could not be fully determined. Hence, the next step in research should be based on prospective longitudinal designs (with a higher sample size and accurate CMDs objective indices collection) to further delineate the protective mechanisms of exercise in reducing the risk of CMDs in PLWH, using both validated exercise questionnaires and objective indices measuring the amount of exercise performed and impact on fitness levels.

## 7. Implications for Practice

Notwithstanding the study limitations, the findings shed some light on the benefits of exercise as a primary prevention intervention, focusing on the risk factors for CMDs in PLWH, as recommended by the European AIDS clinical society [39]. Indeed, exercise is a well-known protective factor against CMDs deaths (HR 0.69, 95% CI: 0.43–1.12) and all-cause mortality (HR 0.64, 95% CI: 0.52–0.79) in the general population [40]. Moreover, in PLWH, recent meta-analyses have shown that exercise significantly improved aerobic capacity, resulting in a better oxygen consumption and 6-minute walk distance [14], as well as strength, where resistance exercise alone or in combination with aerobic exercise improved upper and lower muscle strength [15]. Hence the value of exercise in a primary lifestyle intervention program for PLWH should be considered to decrease the risk for CMDs, following existent exercise guidelines [40], i.e., intensity in METS (moderate: 5.6–6 METS), time (≥150 min), and frequency (at least 1 session·week^−1^) [21].

## 8. Conclusions

The promotion of exercise is warranted to facilitate improvements in cardiovascular and metabolic health in PLWH. However, further studies are needed to elucidate the mechanisms pertaining to exercise as a protective factor in PLWH against CMDs.

## Figures and Tables

**Table 1:** Cohort characteristics.

Parameter	Overall	Sedentary	Exercise
	n = 446	n = 166	n = 280
Gender			
Female, n (%)	31 (7)	17 (10.2)	14 (5) *
Male, n (%)	414 (92.8)	148 (89.2)	266 (95) *
Other, n (%)	3 (0.2)	1 (0.6)	0
Age (years), m (SD)	44(10)	46(11)	43 (9) *
Height (cm)	180 (150–198)	178 (156–197)	180 (150 -198)
Weight (kg)	77 (50–178)	77 (50–138)	77 (52–178)
BMI (kg·m^2−1^)	23.7 (15.7–63)	23.8 (15.7–41.6)	23.6 (17–63)
Years with HIV	8 (1–32)	7 (1–32)	8 (1–32)
ART			
Yes, n (%)	412 (92.4)	150 (90.4)	262 (93.6)
No, n (%)	34 (7.6)	16 (9.6)	18 (6.4)
CD4 cell count category			
Unknown, n (%)	44 (9.9)	17 (10.2)	27 (9.6)
>500 cell/µL, n (%)	264 (59.2)	82 (49.4)	182 (65) *
200–499 cell/µL, n (%)	108 (24.2)	48 (28.9)	60 (21.4)
<200 cell/µL, n (%)	30 (6.7)	19 (11.4)	11 (3.9) *

Note. Data presented in median and interquartile range (IQR), number of participants (n), and percentage (%). Patient self-reported: antiretroviral treatment (ART), CD4 cell count expressed in cell per microliters (cell·µL^−1^). Significant differences at the 0.05 level between PLWH performing exercise or not (*).

**Table 2:** Cohort exercise characteristics.

Exercise Characteristics	n = 280
Frequency (days·week^−1^)	
<2	33 (11.8)
2–3	169 (60.3)
>3	78 (27.9)
Intensity (MET·min·day^−1^)	
≤103	99 (35.3)
104–190	86 (30.7)
>190	95 (34)
Time (h·week^−1^)	
<2	59 (21.1)
2–4	99 (35.4)
>4	122 (43.5)

Note. Data presented in total number of participants and percentage (%).

**Table 3:** Cardiovascular diseases after the HIV diagnosis.

Parameter	Sedentary	Exercise	Total	X^2^	df	*p* Value
	n = 166	n = 280				
Dyslipidemia, n (%)				1.12	2	>0.05°
Pre-HIV	2 (1.2)	1 (0.3)	3			
Post-HIV	4 (2.4)	7 (2.5)	11			
Diabetes, n (%)				0.61	2	>0.05°
Pre-HIV	1(0.6)	0	1			
Post-HIV	2 (1.2)	1 (0.3)	3			
Lipodystrophy, n (%)				0.08	1	>0.05
Post-HIV	4 (2.4)	8 (2.8)	12			
Hypertension, n (%)				0.43	2	>0.05
Pre-HIV	10 (6)	14 (5)	24			
Post-HIV	8 (4.8)	11 (3.9)	19			
Atherosclerosis, n (%)				5.67	2	>0.05°
Pre-HIV	3 (1.8)	0	3			
Post-HIV	0	1 (0.3)	1			
PAD, n (%)				1.69	1	>0.05°
Pre-HIV	0	0	0			
Post-HIV	1 (0.6)	0	1			
Heart failure, n (%)				1.69	1	>0.05°
Pre-HIV	0	0	0			
Post-HIV	0	1 (0.3)	1			
Heart arrhythmias, n (%)				2.87	2	>0.05°
Pre-HIV	1(0.6)	0	1			
Post-HIV	0	2 (0.6)	2			
CHD, n (%)				1.21	1	>0.05°
Pre-HIV	0	0	0			
Post-HIV	2 (1.2)	1 (0.3)	3			
MI, n (%)				0.17	1	>0.05°
Pre-HIV	0	0	0			
Post-HIV	2 (1.2)	3 (60)	5			
Stroke, n (%)				0.59	1	>0.05°
Pre-HIV	0	1 (100)	1			
Post-HIV	0	0	0			
One or more CMD, n (%)						
Pre -HIV	15 (9)	15 (5.3)	30	2.24	1	> 0.05
Post-HIV	13 (7.8)	25 (8.9)	38	0.16	1	>0.05
More than one NCD, n (%)						
Post-HIV	4 (2.4)	4 (1.4)	8	0.56	1	>0.05

Note. Data presented in number of participants (n) and percentage (%), Chi square (X2), degrees of freedom (df), coronary heart disease (CHD), myocardial infarction (MI), peripheral arterial disease (PAD), Fisher’s exact test (°).

**Table 4:** Risk of reporting CMDs in relation to anthropometric characteristics, HIV and ART, EXC characteristics, and exercise in German PLWH.

Variables	Atherosclerosis Post HIV			Hypertension Post HIV			CHD Post HIV			Diabetes Mellitus Post HIV		
	PR	95% CI	*p*	PR	95% CI	*p*	PR	95% CI	*p*	PR	95% CI	*p*
Model 1												
Age (years)	0.9	0.8–1.1	0.43	1	1–1.1	0.21	0.8	0.7–1.1	<0.01	1.1	1–1.2	0.64
Weight (kg)	1	0.8–1.3	0.16	1	0.9–1.1	0.82	1.2	1–1.3	<0.01	1.2	1.2–1.4	<0.01
BMI (kg·m^2−1^)	0.8	0.3–1.6	<0.01	1.1	0.9–1.4	0.14	0.8	0.5–1.1	<0.01	1.1	1–1.1	<0.01
Model 2												
Years since the HIV diagnosis (years)	1.2	1–1.4	<0.01	1.1	1–1.2	< 0.01	1.2	1.1–1.5	<0.01	1.1	1–1.2	0.47
Model 3												
Frequency (session·week^−1^)	0.9	0.4–1.4	0.30	0.7	0.4–1	0.09	1.1	0.7–1.4	0.05	0.4	0.1–1	<0.01
Intensity (MET·min·day^−1^)	1	1 – 1.1	<0.01	1	0.9–1	0.02	1	1–1.1	<0.01	1	0.9–1	0.62
Duration (h·week^−1^)	1	0.9–1.3	<0.01	1	1–1.2	<0.01	1	0.8–1.3	<0.01	1	0.6–1.3	<0.01
Model 4												
Exercise	-	-	-	0.8	0.3–2	0.63	0.3	0.1–3	0.31	1.2	0.1–25.3	0.88
Sedentary	-	-	-	Ref	Ref	Ref	Ref	Ref	Ref	Ref	Ref	Ref
**Variables**	**Dyslipidemia Post HIV**			**Heart Arrhythmias Post HIV**			**Myocardial Infarct Post HIV**			**>1 CMD Post HIV**		
	**PR**	**95% CI**	***p***	**PR**	**95% CI**	***p***	**PR**	**95% CI**	***p***	**PR**	**95% CI**	***p***
Model 1												
Age (years)	1.1	1–1.1	0.01	1.2	1–1.4	<0.01	1	0.9–1.2	0.84	1	1–1.2	0.90
Weight (kg)	1	0.96–1.1	0.50	1.3	1–1.6	<0.01	1	0.0–1.2	0.32	1.1	1–1.2	<0.01
BMI (kg·m^2−1^)	1	0.86–1.2	0.75	0.7	042–1.1	0.03	0.9	0.5–1.5	0.63	0.9	0.6–1.2	0.08
Model 2												
Years since the HIV diagnosis (years)	1.1	1–1.1	0.02	1	0.9–1.2	0.34	1.1	1–1.3	0.08	1.1	1–1.2	0.14
Model 3												
Frequency (session·week^−1^)	1	0.5–1.3	0.81	1.6	0.90–2.5	<0.01	1.2	0.9–1.5	0.05	1	0.6–1.3	0.72
Intensity (MET·min·day^−1^)	1	0.9–1	0.43	1	0.9–1	<0.01	1	0.9–1	0.19	1	1–1.1	0.08
Duration (h·week^−1^)	1	0.8–1.1	0.53	0.2	0.1–0.62	>0.01	1	0.7–1.2	0.80	1.1	0.9–1.2	0.06
Model 4												
Exercise	1	0.3–3.9	0.95	-	-	-	0.9	0.1–6.7	0.90	0.6	0.1–2.5	0.45
Sedentary	Ref	Ref	Ref	-	-	-	Ref	Ref	Ref	Ref	Ref	Ref

Note. Prevalence ratio (PR), 95% confidence interval (95% CI), body mass index (BMI), exercise intensity expressed in metabolic equivalent of task (METs), reference group (Ref), not possible to calculate (-).

## Data Availability

The data presented in this study are available in Table S1: Supplementary table.

## Supplementary Materials

The following are available online at https://www.mdpi.com/article/10.3390/ijerph182111579/s1, Table S1: Supplementary table. Supplementary Questionnaire S1: HIBES questionnaire.
