# Supplementary material for: Recreational Exercising and Self-Reported Cardiometabolic Diseases in German People Living with HIV: A Cross-Sectional Study
Source: Int J Environ Res Public Health. 2021 Nov 4;18(21):11579. doi: 10.3390/ijerph182111579 (PMC8583567; doi:10.3390/ijerph182111579)
Supplement: Supplementary file 1 [file ijerph-18-11579-s001.zip › Supplementary material/S2_HIBES_ questionnaire.pdf]

HUMBOLDT-UNIVERSITÄT ZU BERLIN
PHILOSOPHISCHE FAKULTÄT IV
SPORTMEDIZIN

Teilnehmerinformation: HIBES- Studie

Die derzeitige medikamentöse Therapie (Highly Active AntiRetroviral Treatment - HAART) gilt für die erfolgreiche Behandlung von HIV als unerlässlich. Folglich können Begleiterkrankungen auftreten.
Ist eine Begleiterkrankung diagnostiziert worden bedeutet das, neben der Einnahme der HAART-Medikamente, die Einnahme von wenigstens einem Medikament mehr.

Es bestehen bereits Bewegungs- und Sporttherapien für Herz- Kreislauf-, Stoffwechsel-, Tumor-, neurologische und systemische Gelenkerkrankungen, die eine Minderung der Medikamenteneinnahme, Erhöhung der Alltags-Leistungsfähigkeit und Erhöhung der Lebensqualität ermöglichen.

Theoretische Erkenntnisse und die angewandte Praxis weisen jedoch eine gewisse Diskrepanz auf. Einerseits kann ein HIV-Betroffener aufgrund mehrerer Erkrankungen viel zu viel Angst haben körperlich aktiv zu werden und nicht über das nötige Wissen hinsichtlich möglicher positive Effekte durch sportliche Betätigung verfügen. Andererseits kann es sein, dass der HIV- Betroffene um den positiven Effekt zwar weiß, in der Dosierung und Anwendung des Sports sowie der optimalen Sportart jedoch im Unklaren ist.

Aus diesem Grunde wird im Rahmen der **HIBES- Studie** (**HI**V – **Be**rlin – **S**port- Studie) beabsichtigt, mit Hilfe einer Befragung Grundlagen für eine Sporttherapie für HIV-positive Patienten mit HIV- und medikamentenbedingten Begleiterkrankungen zu schaffen. Auf Grundlage dieser Umfrage soll anschließend ein Praxisratgeber entwickelt werden.

Im Rahmen der Zusammenarbeit mit dem Kooperationspartner **Kompetenznetz HIV / AIDS** (gefördert durch das Bundesministerium für Bildung und Forschung (BMBF)) ist eine Ergänzung der **HIBES- Studie** mit medizinischen Daten des **Kompetenznetz HIV / AIDS** geplant. Diese Kooperation kann dazu beitragen, wichtige Fragen zu Auswirkungen von Sport und Bewegung in der HIV-Therapie zu beantworten. Sollten Sie Teilnehmer am **Kompetenznetz HIV / AIDS** sein, möchten wir Sie deshalb bitten, zu Beginn Ihre PID-Nummer (Pseudonym-Nummer) einzutragen. Sollte Sie diese nicht kennen, kann die PID bei Ihrem behandelnden Arzt erfragt werden.

Die **HIBES-Studie** wurde behördlichen Datenschutzbeauftragten der Humboldt- Universität zu Berlin geprüft und genehmigt. Die Sicherung der Anonymität wurde entsprechend der Richtlinien des Datenschutzes vom Datenschutzbeauftragten der Humboldt- Universität zu Berlin und vom Kompetenznetz HIV / AIDS für gegeben erklärt. Die **HIBES-Studie** wurde des Weiteren von der Ethikkommission der Charité - Universitätsmedizin Berlin - geprüft und genehmigt.

Die **HIBES- Studie** wird unterstützt durch das Kompetenznetz HIV / AIDS, die DAGNÄ, die HIV-Schwerpunktpraxen Deutschland, der Berliner Aidshilfe und deren Initiativen, Print- und Onlinemagazine und der European Gay and Lesbian Sport Federation (EGLSF).

Wir bitten Sie, sich deshalb an der anonymen Befragung zu beteiligen und andere Betroffene darauf hinzuweisen.

☐ Ich habe die Teilnehmerinformation gelesen.

Erläuterungen zum Datenschutz

Ihre Teilnahme ist vollkommen freiwillig. Es entstehen Ihnen keinerlei Nachteile, wenn Sie nicht teilnehmen. Sie können die Teilnahme jederzeit abbrechen.

Die nachfolgenden Fragen sind mit größtmöglicher Sorgfalt so ausgewählt worden, dass eine anonyme Erhebung und Verarbeitung der Daten gewährleistet ist.

Die Befragung beschränkt sich auf bestimmte medizinische und körperliche Merkmale. Es werden weder Name noch (E-Mail-)Adresse abgefragt, noch Ihre IP-Adresse gespeichert. Sollten Sie als Teilnehmer am Kompetenznetz HIV / AIDS Ihre PID eingetragen haben, werden Ihre medizinischen Daten ausschließlich anonymisiert erhoben. Die Datenerhebung erfolgt ausschließlich zur anonymen Auswertung im Rahmen einer Promotionsarbeit. Zugriff auf die erhobenen Daten haben nur Herr Philipp Zech, ferner die am Prüfverfahren beteiligten Betreuer, jedoch nur, soweit dies erforderlich ist. Erhobene Daten werden nicht an außenstehende Dritte bzw. Institutionen weitergegeben bzw. für weitere nicht-wissenschaftliche Zwecke verwendet. Vor Veröffentlichung der Auswertungsergebnisse wird nochmals geprüft, dass keine Rückschlüsse auf Personen möglich sind. Die anonymen Datensätze werden nach den Regeln der Deutschen Forschungsgemeinschaft (DFG) zur „Good Scientific Practice“ nach Beendigung des Forschungsvorhabens gelöscht.

Bei Fragen und Rückmeldungen zum Fragebogen können Sie sich per Mail an Herrn Philipp Zech wenden. Für eine anonyme Kontaktaufnahme können Sie z.B. eine eigens erstellte, unpersönliche E-Mail-Adresse nutzen.

Verantwortlich für die Datenerhebung ist

Herr Philipp Alexander Zech
c/o Humboldt- Universität zu Berlin, Institut für Sportwissenschaften,
Abteilung Sportmedizin
Philippstr. 13, Haus 11,
10115 Berlin

Email: hivsport@gmx.de

☐ Ich habe die Erläuterungen zum Datenschutz gelesen und bin damit einverstanden.

HUMBOLDT-UNIVERSITÄT ZU BERLIN

ABT. SPORTMEDIZIN

Institut für Sportwissenschaft

HIBES – Studie: Fragebogen

1. Allgemeines

1.1 Heutiges Datum: **(Tag):____; (Monat):____; (Jahr):_______**

1.2 Sollten Sie Teilnehmer an der Patienten-Kohorte des **Kompetenznetzes HIV / AIDS** sein und einige der dort dokumentierten klinischen Daten für unsere Studie zur Verfügung stellen wollen (siehe Patienten-Information), vermerken Sie bitte hier das Ihnen für jene Studie zugeteilte **Pseudonym (PID)**. Sollten Sie dieses nicht selbst kennen, fragen Sie bitte Ihren behandelnden Arzt.

PID: ☐☐☐☐☐☐☐☐

1.3 Ihr Wohnort ist in:

Deutschland

Berlin: ☐

Nicht Berlin: ☐

Ausland

Land:______________________

1.4 Wie viele Einwohner hat ihr Wohnort?

bis 100.000: ☐

100.000 – 500.000: ☐

über 500.000: ☐

2. Persönliche Daten und sozioökonomischer Status:

2.1 Körperhöhe in cm: _________ cm

2.2 Körpergewicht in kg: _________ kg

2.3 Ihr Geschlecht:

männlich: ☐

weiblich: ☐

Andere: ☐

2.4 In welchem Jahr wurden Sie geboren:

19______

2.5 Wie ist ihr Familienstand?

Ledig: ☐

Verheiratet: ☐

Geschieden: ☐

Verwitwet: ☐

Der Fragebogen HIBES-Studie einschließlich der Inhalte unterliegt dem Copyright von Philipp Alexander Zech und Dr. med. Lars Brechtel.
Alle Rechte vorbehalten. Wiederverwendung ohne Zustimmung ist nicht gestattet.

2.6 Leben Sie in einer Partnerschaft?

Ja: ☐ Nein: ☐

2.7 In welchem Land wurden Sie geboren?

____

In welchem Land wurde Ihre Mutter geboren?

______________________________________ weiß nicht: ☐

In welchem Land wurde Ihr Vater geboren?

______________________________________ weiß nicht: ☐

2.8 Mit der nächsten Frage möchten wir Sie zu Ihren Selbstbezeichnungen befragen.
Wie beschreiben Sie selbst Ihre ethnische/ kulturelle/ nationale Identität und Zugehörigkeit?

Weiße / Weißer	☐	Filipinisch	☐
Afrikanisch- amerikanisch	☐	Thailändisch	☐
Afrikanisch	☐	Arabisch	☐
Afrikanisch (Coloured)	☐	Chinesisch	☐
Hispanisch / Latino	☐	Japanisch	☐
Mexikanisch	☐	Türkisch	☐
Mexikanisch-amerikanisch (chicano)	☐	Vietnamesisch	☐
Asiatisch-Indisch (Indisch- Amerikanisch)	☐	Asiatisch (Colored)	☐
Karibisch	☐	Indisch	☐
Karibisch (Colored)	☐		

Sollten Ihre Selbstbezeichnungen nicht auf eine der oben genannten ethnischen/ kulturellen/ nationalen Identitäten und Zugehörigkeiten zutreffen, so kreuzen Sie bitte das nachfolgende Kästchen an und tragen Sie Ihre Selbstbezeichnungen ein. Benutzen Sie bitte gegebenenfalls auch Begriffe zur Selbstbezeichnung wie z.B. Schwarze/r, Person of Color, Nichtweiße/r, Weiße/r oder keine.

☐ ____

2.9 Wie viele Personen leben ständig in Ihrem Haushalt, Sie selbst mit eingeschlossen?

Ingesamt ______ Personen

Wie viele davon sind Kinder unter 18 Jahren?

Ingesamt ______ Personen

2.10 Was ist Ihr höchster Schulabschluss?
(nicht gemeint sind Hochschulabschlüsse wie z.B. Studium etc…)

Hauptschule: ☐
Abitur/ allgemeine Hochschulreife: ☐
Anderer Schulabschluss: ☐
Realschule/ Mittlere Reife: ☐
Fachhochschulreife: ☐
Kein Schulabschluss: ☐

Der Fragebogen HIBES-Studie einschließlich der Inhalte unterliegt dem Copyright von Philipp Alexander Zech und Dr. med. Lars Brechtel.
Alle Rechte vorbehalten. Wiederverwendung ohne Zustimmung ist nicht gestattet.

Wie viele Jahre haben Sie die Schule besucht?

< 8 Jahre:	☐
8 - 9 Jahre:	☐
10 Jahre:	☐
11 – 13 Jahre:	☐

2.11 Welchen beruflichen Ausbildungsabschluss haben Sie?
(Mehrfachnennungen sind möglich)

Lehre:	☐
Fachhochschule, Ingenieurschule:	☐
Universität, Hochschule:	☐
Andere Berufausbildung:	☐
Keine Berufsausbildung:	☐

2.12 Sind Sie zurzeit erwerbstätig?

Ja,	ganztägig:	☐
	mindestens halbtags:	☐
	weniger als halbtags:	☐
Nein,	Hausfrau/ Hausmann:	☐
	in Ausbildung/ Umschulung:	☐
	arbeitslos/ erwerbslos:	☐
	Erwerbs-, Berufsunfähigkeitsrente:	☐
	Altersrente:	☐
	Anderes:	☐

2.13 In welcher beruflichen Stellung sind Sie zurzeit bzw. waren Sie zuletzt beschäftigt?

Arbeiter:	☐
Angestellter:	☐
Beamter:	☐
Selbstständiger:	☐
Sonstiges:	☐

2.14 Wie hoch ist das monatliche Nettoeinkommen Ihres Haushaltes insgesamt?
(Nettoeinkommen: Summe aus Lohn/ Gehalt/ Einkommen usw. jeweils unter Abzug der Steuern und Sozialabgaben)

Bis unter 500 Euro:	☐
500 - 999 Euro:	☐
1000 - 1499 Euro:	☐
1500 - 1999 Euro:	☐
2000 - 2499 Euro:	☐
2500 - 2999 Euro:	☐
3000 - 3499 Euro:	☐
3500 Euro und mehr:	☐

2.15 Wie viele Stunden pro Woche arbeiten Sie?

_________ Stunden

Der Fragebogen HIBES-Studie einschließlich der Inhalte unterliegt dem Copyright von Philipp Alexander Zech und Dr. med. Lars Brechtel.
Alle Rechte vorbehalten. Wiederverwendung ohne Zustimmung ist nicht gestattet.

3. HIV und Medikamenteneinnahme

3.1 Ist bei Ihnen HIV diagnostiziert worden?

Ja: ☐ Nein: ☐

3.2 Wann ist bei Ihnen HIV diagnostiziert worden?

Monat: _______________ Jahr:________________

3.3 Ist Ihnen Ihr **aktueller** CD4- T- Helferzellwert bekannt?

Ja: ☐ Nein: ☐

Wenn „**Ja**“, wie hoch ist er?

Ungefähre Angabe:

0 – 50	CD4- Zellen/ µL	☐
51 – 99	CD4- Zellen/ µL	☐
100 – 199	CD4- Zellen/ µL	☐
200 – 499	CD4- Zellen/ µL	☐
> 500	CD4- Zellen/ µL	☐

Falls Sie Ihren exakten **aktuellen** CD4- Helferzellwert kennen, bitte notieren Sie ihn hier: ___________

3.4 Ist Ihnen Ihr **niedrigster** CD4- T- Helferzellwert bekannt?
(Den niedrigsten CD4- Helferzellwert, den Sie jemals hatten)

Ja: ☐ Nein: ☐

Wenn „**Ja**“, wie hoch ist er?

Ungefähre Angabe:

0 – 50	CD4- Zellen/ µL	☐
51 – 99	CD4- Zellen/ µL	☐
100 – 199	CD4- Zellen/ µL	☐
200 – 499	CD4- Zellen/ µL	☐
> 500	CD4- Zellen/ µL	☐

Falls Sie Ihren exakten **niedrigsten** CD4- Helferzellwert kennen, bitte notieren Sie ihn hier: ___________

Der Fragebogen HIBES-Studie einschließlich der Inhalte unterliegt dem Copyright von Philipp Alexander Zech und Dr. med. Lars Brechtel.
Alle Rechte vorbehalten. Wiederverwendung ohne Zustimmung ist nicht gestattet.

3.5 Ist Ihnen Ihre **aktuelle** Viruslast bekannt?

Ja: ☐ Nein: ☐

Wenn „**Ja**", wie hoch ist sie?

Ungefähre Angaben:

**Unter der Nachweisgrenze**		☐
50 – 99	copies/ ml	☐
100 - 1.000	copies/ ml	☐
1.000 – 10.000	copies/ ml	☐
10.000 – 50.000	copies/ ml	☐
50.000 – 100.000	copies/ ml	☐
1000.000 – 500.000	copies/ ml	☐
500.000 – 1.000.000	copies/ ml	☐
> 1.000.000	copies/ ml	☐

Falls Sie Ihre exakte **aktuelle** Viruslast kennen, bitte notieren Sie sie hier:

______

3.6 Nehmen Sie zurzeit gegen die HIV- Erkrankung anti-retrovirale HIV- Medikamente (ART/ HAART) ein?

Ja: ☐

Nein: ☐
Wenn „**Nein**" **bitte weiter mit Frage 3.7**

Wenn „**Ja**", befindet sich a**uf der <u>nächsten</u> Seite (Extrablatt: - Medikamentenliste-) eine Auflistung der Medikamente. Bitte tragen Sie das Datum des Beginns (Monat / Jahr) der aktuellen Medikamenteneinnahme ein und kreuzen Sie die derzeit eingenommenen Medikamente an (Kästchen). Danach machen Sie bitte mit Frage 3.7 weiter!**

Der Fragebogen HIBES-Studie einschließlich der Inhalte unterliegt dem Copyright von Philipp Alexander Zech und Dr. med. Lars Brechtel.
Alle Rechte vorbehalten. Wiederverwendung ohne Zustimmung ist nicht gestattet.

-Medikamentenliste-

Bitte hier das Datum des Beginns der aktuellen Therapie eintragen:

Aktuelle Therapie
seit
Monat / Jahr

______ /______

Bitte kreuzen Sie Ihre derzeit eingenommenen Medikamente an:

Handelsname	Wirkstoff	Abkürzung	
**Kombinationspräparate**			
Atripla	Tenofovir, Emtricitabin und Efavirenz	TDF, FTC und EFV	☐
Combivir	Lamivudin und Zidovudin	3TC und AZT	☐
Kivexa	Lamivudin und Abacavir	3TC und ABC	☐
Epzicom	Lamivudin und Abacavir	3TC und ABC	☐
Trizivir	Lamivudin, Abacavir und Zidovudin	3TC, ABC und AZT	☐
Truvada	Tenofovir und Emtricitabin	TDF und FTC	☐
**Nukleosidanaloge/ Nukleotidanaloge Reverse-Transkriptase-Inhibitoren (NRTI)**			
Emtriva	Emtricitabin	FTC	☐
Epivir	Lamivudin	3TC	☐
Retrovir	Zidovudin	AZT	☐
Videx	Didanosin	ddI	☐
Viread	Tenofovir	TDF	☐
Zerit	Stavudin	d4T	☐
Ziagen	Abacavir	ABC	☐
Hivid	Zalcitabin	ddC	☐
**Nicht-Nukleosidale Reverse-Transkriptase-Inhibitoren (NNRTI)**			
Intelence	Etravirin	ETV	☐
Sustiva	Efavirenz	EFV	☐
Stocrin	Efavirenz (amerikanisches Präparat)	EFV	☐
Viramune	Nevirapin	NVP	☐
Rescriptor	Delavirdin	DVD	☐
**Protease-Inhibitoren (PI)**			
Aptivus	Tipranavir	TPV	☐
Crixivan	Indinavir	IDV	☐
Invirase	Saquinavir	SQV	☐
Kaletra	Lopinavir und Ritonavir	LPV	☐
Prezista	Darunavir	DRV	☐
Reyataz	Atazanavir	ATV	☐
Telzir	Fosamprenavir	FPV	☐
Lexiva	Fosamprenavir (amerikanisches Präparat)	FPV	☐
Viracept	Nelfinavir	NFV	☐
Fortovase	Saquinavir	SQV	☐
Agenerase	Amprenavir	APV	☐
**Entry-Inhibitoren**			
Fuzeon	Enfuvirtide	ENF	☐
Celsentri	Maraviroc	MVC	☐
Selzentry	Maraviroc (amerikanisches Präparat)	MVC	☐
**Integrase-Inhibitoren**			
Isentress	Raltegravir	RAL	☐
Sonstige			

Der Fragebogen HIBES-Studie einschließlich der Inhalte unterliegt dem Copyright von Philipp Alexander Zech und Dr. med. Lars Brechtel.
Alle Rechte vorbehalten. Wiederverwendung ohne Zustimmung ist nicht gestattet.

3.7 Haben Sie vorher schon andere anti-retrovirale HIV- Medikamente (ART / HAART) eingenommen?

Ja: ☐ Nein: ☐

3.8 Seit wann nehmen Sie überhaupt anti-retrovirale **HIV-** Medikamente ein (allererster Therapiebeginn)?

Monat:________________ Jahr:_________________

3.9 Wie oft wurde die anti- retrovirale Therapie/**HIV-** Medikamente gewechselt?

______mal

4. Begleiterkrankungen

4.1 Haben Sie oder hatten Sie neben Ihrer HIV- Erkrankung eine weitere Erkrankung (siehe Liste der Begleiterkrankungen in Frage 4.2)?
Dazu zählen z.B. Bluthochdruck, Lipodystrophie, Diabetes, Übergewicht, Pilzinfektionen, virale Infektionen, bakterielle Infektionen, Tumore (Krebs), psychische Erkrankungen, sexuell übertragbare Erkrankungen. (Also eigentlich alles außer Husten, Schnupfen und Halsweh)

Ja: ☐ ↓

Aktuelle weitere Erkrankung: ☐ ↓

Weitere Erkrankung(en) in der Vergangenheit: ☐ ↓

Bitte machen Sie weiter mit Frage 4.2.

Noch nie eine weitere Erkrankung: ☐ ↓

Wenn „Noch nie“, bitte machen Sie weiter mit Frage 4.4

Der Fragebogen HIBES-Studie einschließlich der Inhalte unterliegt dem Copyright von Philipp Alexander Zech und Dr. med. Lars Brechtel. Alle Rechte vorbehalten. Wiederverwendung ohne Zustimmung ist nicht gestattet.

4.2 In der folgenden Tabelle befindet sich eine Auflistung von Begleiterkrankungen. Bitte kreuzen Sie an, ob die Erkrankung VOR oder NACH der HIV- INFEKTION aufgetreten ist und welche dieser Begleiterkrankungen Sie AKTUELL haben.
Mehrfachnennungen sind also möglich. *(Es kann beispielsweise sein, dass eine Erkrankung Vor und Nach der HIV-Infektion vorhanden war und **immer noch** aktuell ist. Oder: Es trat eine Erkrankung nach der HIV- Infektion auf, besteht aber JETZT nicht mehr.*

Bitte **KREUZEN** Sie an!

**Begleiterkrankungen**	**Auftreten vor der HIV- Infektion**	**Auftreten nach der HIV- Infektion**	**Besteht noch aktuell**
Fettstoffwechselstörung (Dyslipidämie)			
Diabetes mellitus (Blutzuckererkrankung)			
Bluthochdruck (Arterielle Hypertonie)			
Arteriosklerose (Arterienverkalkung)			
Arterielle, periphere Verschlusserkrankung (pAVK; Schaufensterkrankheit)			
Herzinsuffizienz (Herzschwäche)			
Herzrhythmusstörung			
Koronare Herzkrankheit (Arteriosklerose der Herzkranzgefäße)			
Herzinfarkt (Myokardinfarkt)			
Schlaganfall (Apoplex, Insult)			
Osteoporose (Knochenschwund, Minderung der Knochendichte)			
Osteopenie (Vorstufe der Osteoporose)			
Lipodystrophie (Fettverteilungsstörung)			
Übergewicht bzw. Adipositas			

**Pilzinfektionen (Candidosen)**			
Pilzinfektion im Mundbereich (oropharyngeale Candidose)			
Soor (Pilzinfektion der Atemwege oder Speiseröhre)			
Ösophagitis (Pilz- oder virale Infektion der Speiseröhre)			
Lungenentzündung (Pneumonie) 2 mal oder mehr innerhalb von 12 Monaten			
Histoplasmose, chronisch (Pilzinfektion, zum Teil mit Gewebeschäden)			
Instestinale Isosporiasis			
Talfieber, Wüstenfieber (Kokzidiodomykose extrapulmonal/ disseminiert)			
Kryptokokkose, extrapulmonal (in der Lunge)			
Kryptokokkose, chronisch (Meningitis)			
PCP Lungenentzündung (Pneumocytose)			

**Virale Infektionen**			
Gürtelrose (Herpes Zoster)			
CMV- Infektionen (Zytomegalievirus-Infektion; außer Leber, Milz, Lymphknoten)			
CMV- Retinitis (Zytomegalievirus-Infektion der Nezthaut mit Einschränkung der Sehschärfe)			
Herpes mit chronischen Geschwüren (Herpes simplex)			
Lungenentzündung (Pneumonie) 2 mal oder mehr innerhalb von 12 Monaten			
Hirnhautenzündung (Meningitis)			
Progressive multifokale Leukenzephalopathie (fortschreitende Erkrankung des zentralen Nervensystems)			
Orale Haarleukoplakie (weiß-streifige Veränderung der Zunge)			

**Bakterielle Infektionen**			
Bazilläre Angiomatose (Geschwülste auf der Haut oder auch Organen)			
Listeriose (Infektion mit Listerien)			
Extrapulmonale Mykobakterien			
Wiederkehrende Lungenentzündung (bakterielle rezidivierende Pneumonie über 1 Jahr)			
Salmonelleninfektionen (rezidivierende Salmonellen-Septikämie = wiederkehrende Gesamtinfektion des Körpers mit Auftreten von Salmonellen im Blut)			
Schwindsucht (Tuberkulose, pulmonal und extrapulmonal)			
MAC (Mycobacterium Avium Complex – Infektion)			

Der Fragebogen HIBES-Studie einschließlich der Inhalte unterliegt dem Copyright von Philipp Alexander Zech und Dr. med. Lars Brechtel. Alle Rechte vorbehalten. Wiederverwendung ohne Zustimmung ist nicht gestattet.

Bitte **KREUZEN** Sie an!

**Begleiterkrankungen**	**Auftreten vor der HIV- Infektion**	**Auftreten nach der HIV- Infektion**	**Auftreten aktuell**
**Tumore, Krebs**			
Karzinoma in situ (Frühstadium eines epithelialen Tumors)			
Kaposi- Sarkom			
Zerebrales Burkitt-Lymphom			
Burkitt-Lymphom			
Non-Hodgekin-Lymphom (NHL)			
Hodgekin- Lymphom (HL)			
Analkarzinom			
**Andere Erkrankungen**			
Thrombozytopenische Purpura (Kapilläre (fleckförmige) Einblutungen in Gehirn, Niere und Haut)			
Fieber/ Durchfall länger als ein Monat			
Periphere Neuropathie (Erkrankung des peripheren Nervensystems)			
HIV- bedingte Enzephalopathie (krankhafte Veränderung des Gehirns)			
Zerebrale Toxoplasmose (parasitäre Infektion des Gehirns mit Toxoplasmen)			
Wasting - Syndrom (ungewollter Gewichtsverlust)			
Leishmaniose (parasitäre Infektion mit Leishmanien)			
**Neurologische und psychiatrische Erkrankungen**			
Polyneuropathie (Erkrankung des peripheren Nervensystems)			
Depression			
Burnout- Syndrom			
Schlafstörungen			
Angststörungen			
Psychosen			
Demenz			
**Erkrankungen der Frau (Gynäkologische Erkrankungen)**			
Adnexitis (Entzündung des Eierstöcke und / oder der Eileiter)			
Entzündung des kleinen Beckens			
Vaginale Candidose (Pilzinfektion der Vagina)			
Zervikale Dysplasien (dysplastische oder abnorme Zellveränderungen des Gebährmutterhales)			
Invasives Zervix- Karzinom (Gebährmutterhalskrebs)			
**Sexuell übertragbare Krankheiten (Geschlechtskrankheiten/ STDs)**			
Chlamydien			
Feigwarzen			
Filzläuse			
Hepatitis B			
Hepatitis C			
Krätze			
Syphilkis			
Trichomoniasis			
Tripper (Gonorrhoe)			
Herpes Genitalis			

Der Fragebogen HIBES-Studie einschließlich der Inhalte unterliegt dem Copyright von Philipp Alexander Zech und Dr. med. Lars Brechtel.
Alle Rechte vorbehalten. Wiederverwendung ohne Zustimmung ist nicht gestattet.

4.3 Nehmen Sie zurzeit gegen Ihre aktuelle Begleiterkrankung zusätzlich Medikamente ein?
(z.B. Bluthochdruckmedikamente wie Betablocker… etc.)

Ja: ☐ Nein: ☐

⇩

Wenn „**Ja**“, welche zusätzlichen Medikamente?

____

4.4 Zu welchem Grade wird das Auftreten folgender Begleiterkrankungen **Ihrer Meinung nach** durch die Einnahme antiretroviraler **HIV-** Medikamente verursacht oder begünstigt?
Kreuzen Sie an:
*Auftreten der Begleiterkrankung aufgrund der antiretroviralen **HIV-** Medikamente*
sehr wahrscheinlich** bis hin zu **überhaupt nicht wahrscheinlich

	**Auftreten Durch HIV-Medikamente sehr wahrscheinlich**	**Auftreten durch HIV-Medikamente wahrscheinlich**	**Es könnte beides sein**	**Auftreten durch HIV-Medikamente unwahrscheinlich**	**Auftreten durch HIV-Medikamente sehr unwahrscheinlich**
Fettstoffwechselstörung (Dyslipidämie)					
Diabetes mellitus (Blutzuckererkrankung)					
Bluthochdruck (Arterielle Hypertonie)					
Arteriosklerose (Arterienverkalkung)					
Arterielle periphere Verschlusserkrankung (pAVK; Schaufensterkrankheit)					
Herzinsuffizienz (Herzschwäche)					
Herzrhythmusstörung					
Koronare Herzkrankheit (Arteriosklerose der Herzkranzgefäße)					
Herzinfarkt (Myokardinfarkt)					
Schlaganfall (Apoplex, Insult)					
Osteoporose (Knochenschwund, Minderung der Knochendichte)					
Osteopenie (Vorstufe der Osteoporose)					
Polyneuropathie (Erkrankung des peripheren Nervensystems)					
Lipodystrophie (Fettverteilungsstörung)					
Übergewicht bzw. Adipositas					
Depression					
Burn-out					
Schlafstörungen					
Angst					
Psychosen					
Demenz					

Der Fragebogen HIBES-Studie einschließlich der Inhalte unterliegt dem Copyright von Philipp Alexander Zech und Dr. med. Lars Brechtel.
Alle Rechte vorbehalten. Wiederverwendung ohne Zustimmung ist nicht gestattet.

5. Sportverhalten von HIV- positiven Patienten

5.1 Treiben Sie aktuell Sport oder haben Sie Sport betrieben?

Ja: ☐ ⇩

Nein: ☐
Wenn „Nein“, **bitte weiter mit Frage 5.4**

5.2 **Nachfolgend befindet sich eine Tabelle „Sport früher und aktuell“. Dort tragen Sie bitte Ihre betriebene Sportart, den Zeitraum, die Häufigkeit pro Woche (Trainingseinheiten) und die Trainingsstunden pro Woche ein. Die Spalte „Regelmäßigkeit“ wird lediglich mit einem „Ja“ oder „Nein“ beantwortet. „Regelmäßig“ bedeutet, wenn mindestens einmal pro Woche Sport über längeren Zeitraum (1 Jahr) betrieben wurde.**

Mehrfachnennungen sind möglich!

Sport aktuell: Bitte tragen Sie Ihre **aktuelle** sportliche Aktivität ein:

Sportart aktuell	Zeitraum (bitte Jahreszahlen angeben)		Häufigkeit (Trainingseinheiten) pro Woche	Trainingsstunden pro Woche	Regelmäßig? Ja / Nein
	von	bis			

Sport früher: Bitte tragen Sie Ihre **früheren** sportlichen Aktivitäten ein:

Sportart früher	Zeitraum (bitte Jahreszahlen angeben)		Häufigkeit (Trainingseinheiten) pro Woche	Trainingsstunden pro Woche	Regelmäßig? Ja / Nein
	von	bis			

Der Fragebogen HIBES-Studie einschließlich der Inhalte unterliegt dem Copyright von Philipp Alexander Zech und Dr. med. Lars Brechtel.
Alle Rechte vorbehalten. Wiederverwendung ohne Zustimmung ist nicht gestattet.

5.3 Hat sich ihr Sportverhalten nach der HIV- Infektion verändert?

Ja: ☐ ⇩

Nein: ☐
Wenn „Nein“, **bitte weiter mit Frage 5.4**

Wenn „Ja“, in wiefern? (*Mehrfachantworten sind möglich!)*

„Seit Bekanntwerden der HIV- Infektion treibe ich mehr Sport als vorher“ ☐
„Seit Bekanntwerden der HIV- Infektion treibe ich weniger Sport als vorher“ ☐
„Seit Bekanntwerden der HIV-Infektion treibe ich genauso viel Sport wie vorher“ ☐
„Nach Bekanntwerden der HIV-Infektion habe ich mit Sport aufgehört“ ☐
„Nach Bekanntwerden der HIV-Infektion habe ich mit Sport begonnen“ ☐
„Nach Bekanntwerden der HIV-Infektion treibe ich einen anderen Sport“ ☐

5.4 Wie schätzen Sie folgende Aussagen ein?
Bitte **kreuzen** Sie von Trifft voll zu bis Trifft überhaupt nicht zu an!

**Bitte Ankreuzen!**	**Trifft voll zu**	**Trifft teilweise zu**	**Weder - noch**	**Trifft weniger zu**	**Trifft überhaupt nicht zu**
„Ich habe Spaß am Sport“					
„Sport laugt mich aus“					
„Ich habe keine Zeit für Sport“					
„Ich habe Kenntnis über eine gesundheitliche Wirkung von Sport auf HIV“					
„Ich habe Hemmungen aufgrund meiner HIV-Infektion Sport zu betreiben“					
„Durch Sport verbessere ich mein Aussehen“					
„Sport schadet meiner Gesundheit“					
„Durch Sport habe ich soziale Kontakte“					
„Eine auf die HIV- Erkrankung abgestimmte Sporttherapie erachte ich als sinnvoll“					
„Unter fachkompetenter HIV-zielgerichteter sportlicher Betreuung würde ich mehr Sport treiben“					
„Vor Sport habe ich Angst“					
„In Fragen zu Sport bei HIV kenne ich mich aus“					
„Wegen gesundheitlicher Probleme treibe ich weniger oder kein Sport“					
„Ich treibe genauso viel Sport wie schon immer“					
„Ich treibe weniger Sport als früher“					
„Sport fördert meine Gesundheit“					
„Sport entspannt mich“					
„Ich habe Kenntnis über die positive Wirkung von Sport auf eine entsprechende Begleiterkrankung“					
„Eine auf die Begleiterkrankung abgestimmte Sporttherapie erachte ich als sinnvoll“					
„Unter fachkompetenter, zielgerichteter sportlicher Betreuung und Berücksichtigung meiner Begleiterkrankung(en) würde ich mehr Sport machen“					
„Ich habe Hemmungen aufgrund meiner Begleiterkrankung(en) Sport zu treiben“					
**Die nächsten beiden Aussagen bitte nur ausfüllen, wenn eine Begleiterkrankung besteht:**					
„Unter fachkompetenter, zielgerichteter sportlicher Betreuung und Berücksichtigung meiner Begleiterkrankung(en) würde ich mehr Sport machen“					
„Ich habe Hemmungen aufgrund meiner Begleiterkrankung(en) Sport zu treiben“					

Der Fragebogen HIBES-Studie einschließlich der Inhalte unterliegt dem Copyright von Philipp Alexander Zech und Dr. med. Lars Brechtel.
Alle Rechte vorbehalten. Wiederverwendung ohne Zustimmung ist nicht gestattet.

Bitte beantworten Sie die folgenden Fragen, indem Sie das entsprechende Kästchen ankreuzen.

1. Wie würden Sie Ihren Gesundheitszustand im Allgemeinen beschreiben?

(Bitte kreuzen Sie nur ein Kästchen an)

Ausgezeichnet........ 1 ☐

Sehr gut........ 2 ☐

Gut .... 3 ☐

Weniger gut........ 4 ☐

Schlecht .... 5 ☐

2. Wie stark waren Ihre Schmerzen in den **letzten 4 Wochen**?

(Bitte kreuzen Sie nur ein Kästchen an)

Ich hatte keine Schmerzen .... 1 ☐

Sehr leicht .... 2 ☐

Leicht........ 3 ☐

Mittelmäßig........ 4 ☐

Stark........ 5 ☐

Sehr stark........ 6 ☐

3. Inwieweit haben **die Schmerzen** Sie in den **vergangenen 4 Wochen** bei der Ausübung Ihrer Alltagstätigkeiten zu Hause und im Beruf behindert?

(Bitte kreuzen Sie nur ein Kästchen an)

Überhaupt nicht........ 1 ☐

Ein bißchen .... 2 ☐

Mittelmäßig........ 3 ☐

Ziemlich........ 4 ☐

Sehr........ 5 ☐

Copyright © 1992 Health Assessment Lab.
All Rights Reserved.
(German MOS-HIV Questionnaire Version 2.1)

4. Im Folgenden sind einige Tätigkeiten beschrieben, die Sie vielleicht an einem normalen Tag ausüben. **Sind Sie durch Ihren derzeitigen Gesundheitszustand** bei diesen Tätigkeiten **eingeschränkt**? Wenn ja, wie stark?

(Bitte kreuzen Sie in jeder Zeile nur ein Kästchen an)

		**Ja, stark eingeschränkt**	**Ja, etwas eingeschränkt**	**Nein, überhaupt nicht eingeschränkt**
		1	2	3
a.	**Anstrengende Tätigkeiten** wie schwere Gegenstände heben, schnell laufen, anstrengenden Sport treiben	☐	☐	☐
b.	**Mittelschwere Tätigkeiten**, wie einen Tisch verschieben, Einkaufstaschen tragen oder kegeln	☐	☐	☐
c.	Bergan gehen oder ein paar Treppenabsätze steigen	☐	☐	☐
d.	Sich beugen, etwas vom Boden aufheben, bücken	☐	☐	☐
e.	Von einer Straßenecke zur nächsten zu Fuß gehen	☐	☐	☐
f.	Essen, sich anziehen, sich baden oder die Toilette benutzen	☐	☐	☐

5. Hindert Sie Ihr Gesundheitszustand daran, Ihren Beruf auszuüben, Arbeiten zu Hause zu verrichten oder einer Ausbildung nachzugehen?

(Bitte kreuzen Sie nur ein Kästchen an)

Ja .... 1 ☐

Nein.. 2 ☐

6. Waren Sie wegen Ihres Gesundheitszustandes gänzlich unfähig, bestimmte Arten von Arbeiten im Beruf, Haushalt oder Ausbildung zu verrichten?

(Bitte kreuzen Sie nur ein Kästchen an)

Ja .... 1 ☐

Nein.. 2 ☐

Copyright © 1992 Health Assessment Lab.
All Rights Reserved.
(German MOS-HIV Questionnaire Version 2.1)

Bitte kreuzen Sie bei jeder Frage die Antwort an, die Ihrem Befinden in den letzten 4 Wochen am ehesten entspricht.

(Bitte kreuzen Sie in jeder Zeile nur ein Kästchen an)

	Immer<br>1	Meistens<br>2	Ziemlich oft<br>3	Manchmal<br>4	Selten<br>5	Nie<br>6
7. Wie oft in den letzten 4 Wochen hat Ihr **Gesundheitszustand Ihre sozialen Kontakte eingeschränkt** (wie Besuche bei Freunden oder Familienangehörigen)?	☐	☐	☐	☐	☐	☐
8. Wie oft in den letzten 4 Wochen:						
a. Waren Sie sehr nervös?	☐	☐	☐	☐	☐	☐
b. Waren Sie ruhig und gelassen?	☐	☐	☐	☐	☐	☐
c. Waren Sie entmutigt und traurig?	☐	☐	☐	☐	☐	☐
d. Waren Sie glücklich?	☐	☐	☐	☐	☐	☐
e. Waren Sie so niedergeschlagen, daß Sie nichts aufheitern konnte?	☐	☐	☐	☐	☐	☐

Copyright © 1992 Health Assessment Lab.
All Rights Reserved.
(German MOS-HIV Questionnaire Version 2.1)

(Bitte kreuzen Sie in jeder Zeile nur ein Kästchen an)

	Immer<br>1	Meistens<br>2	Ziemlich oft<br>3	Manchmal<br>4	Selten<br>5	Nie<br>6
9. Wie oft in den **letzten 4 Wochen**:						
a. Waren Sie voller Schwung?	☐	☐	☐	☐	☐	☐
b. Fühlten Sie sich erschöpft?	☐	☐	☐	☐	☐	☐
c. Fühlten Sie sich müde?	☐	☐	☐	☐	☐	☐
d. Hatten Sie genug Energie, die Dinge zu tun, die Sie tun wollten?	☐	☐	☐	☐	☐	☐
e. Fühlten Sie sich durch Ihre gesundheitlichen Probleme belastet?	☐	☐	☐	☐	☐	☐
f. Waren Sie wegen Ihrer gesundheitlichen Probleme entmutigt?	☐	☐	☐	☐	☐	☐
g. Fühlten Sie sich wegen Ihrer gesundheitlichen Probleme verzweifelt?	☐	☐	☐	☐	☐	☐
h. Hatten Sie wegen Ihres Gesundheitszustands Angst?	☐	☐	☐	☐	☐	☐

Copyright © 1992 Health Assessment Lab.
All Rights Reserved.
(German MOS-HIV Questionnaire Version 2.1)

(Bitte kreuzen Sie in jeder Zeile nur ein Kästchen an)

	Immer	Meistens	Ziemlich oft	Manchmal	Selten	Nie
	1	2	3	4	5	6
10. Wie oft in den **letzten 4 Wochen**						
a. Hatten Sie Schwierigkeiten beim Nachdenken und beim Lösen von Problemen, z.B. beim Planen, Entscheiden, Lernen neuer Dinge?	☐	☐	☐	☐	☐	☐
b. Haben Sie Dinge vergessen, die vor kurzem passiert sind, beispielsweise den Ort, an dem Sie etwas abgelegt hatten, oder eine Verabredung?	☐	☐	☐	☐	☐	☐
c. Hatten Sie Schwierigkeiten, Ihre Aufmerksamkeit über einen längeren Zeitraum auf eine Tätigkeit zu halten?	☐	☐	☐	☐	☐	☐
d. Hatten Sie Schwierigkeiten, Tätigkeiten auszuführen, die Konzentration und Nachdenken erfordern?	☐	☐	☐	☐	☐	☐

(Bitte kreuzen Sie in jeder Zeile nur ein Kästchen an)

	Trifft ganz zu	Trifft weitgehend zu	Weiß nicht	Trifft weitgehend nicht zu	Trifft überhaupt nicht zu
	1	2	3	4	5
11. Bitte kreuzen Sie für jede Aussage an, wie zutreffend oder unzutreffend sie für Sie ist.					
a. Ich bin etwas kränklich.	☐	☐	☐	☐	☐
b. Ich bin genauso gesund wie alle anderen, die ich kenne.	☐	☐	☐	☐	☐
c. Ich erfreue mich ausgezeichneter Gesundheit.	☐	☐	☐	☐	☐
d. Ich habe mich in letzter Zeit schlecht gefühlt.	☐	☐	☐	☐	☐

Copyright © 1992 Health Assessment Lab.
All Rights Reserved.
(German MOS-HIV Questionnaire Version 2.1)

12. Wie war Ihre Lebensqualität, d.h. wie ging es Ihnen in den **letzten 4 Wochen**?

(Bitte kreuzen Sie nur ein Kästchen an)

Sehr gut, könnte nicht besser sein.. 1 ☐

Ziemlich gut.. 2 ☐

Mal gut, mal schlecht .... 3 ☐

Ziemlich schlecht .... 4 ☐

Sehr schlecht, könnte nicht schlechter sein........................... 5 ☐

13. Im Vergleich zu **vor 4 Wochen**, wie würden Sie Ihren derzeitigen Gesundheitszustand und Ihre seelische Verfassung beschreiben?

(Bitte kreuzen Sie nur ein Kästchen an)

Derzeit viel besser.. 1 ☐

Derzeit etwas besser.. 2 ☐

Etwa gleich.. 3 ☐

Derzeit etwas schlechter .... 4 ☐

Derzeit viel schlechter .... 5 ☐

Copyright © 1992 Health Assessment Lab.
All Rights Reserved.
(German MOS-HIV Questionnaire Version 2.1)

14. Bitte kreuzen Sie unter jeder Überschrift DAS Kästchen an, das Ihre Gesundheit HEUTE am besten beschreibt.

BEWEGLICHKEIT / MOBILITÄT

Ich habe keine Probleme, herumzugehen ❑
Ich habe leichte Probleme, herumzugehen ❑
Ich habe mäßige Probleme, herumzugehen ❑
Ich habe große Probleme, herumzugehen ❑
Ich bin nicht in der Lage, herumzugehen ❑

FÜR SICH SELBST SORGEN

Ich habe keine Probleme, mich selbst zu waschen oder anzuziehen ❑
Ich habe leichte Probleme, mich selbst zu waschen oder anzuziehen ❑
Ich habe mäßige Probleme, mich selbst zu waschen oder anzuziehen ❑
Ich habe große Probleme, mich selbst zu waschen oder anzuziehen ❑
Ich bin nicht in der Lage, mich selbst zu waschen oder anzuziehen ❑

ALLTÄGLICHE TÄTIGKEITEN *(z. B. Arbeit, Studium, Hausarbeit, Familien- oder Freizeitaktivitäten)*

Ich habe keine Probleme, meinen alltäglichen Tätigkeiten nachzugehen ❑
Ich habe leichte Probleme, meinen alltäglichen Tätigkeiten nachzugehen ❑
Ich habe mäßige Probleme, meinen alltäglichen Tätigkeiten nachzugehen ❑
Ich habe große Probleme, meinen alltäglichen Tätigkeiten nachzugehen ❑
Ich bin nicht in der Lage, meinen alltäglichen Tätigkeiten nachzugehen ❑

SCHMERZEN / KÖRPERLICHE BESCHWERDEN

Ich habe keine Schmerzen oder Beschwerden ❑
Ich habe leichte Schmerzen oder Beschwerden ❑
Ich habe mäßige Schmerzen oder Beschwerden ❑
Ich habe starke Schmerzen oder Beschwerden ❑
Ich habe extreme Schmerzen oder Beschwerden ❑

ANGST / NIEDERGESCHLAGENHEIT

Ich bin nicht ängstlich oder deprimiert ❑
Ich bin ein wenig ängstlich oder deprimiert ❑
Ich bin mäßig ängstlich oder deprimiert ❑
Ich bin sehr ängstlich oder deprimiert ❑
Ich bin extrem ängstlich oder deprimiert ❑

Germany (German) v.2 © 2010 EuroQol Group. EQ-5D™ is a trade mark of the EuroQol Group

Beste Gesundheit, die Sie sich vorstellen können

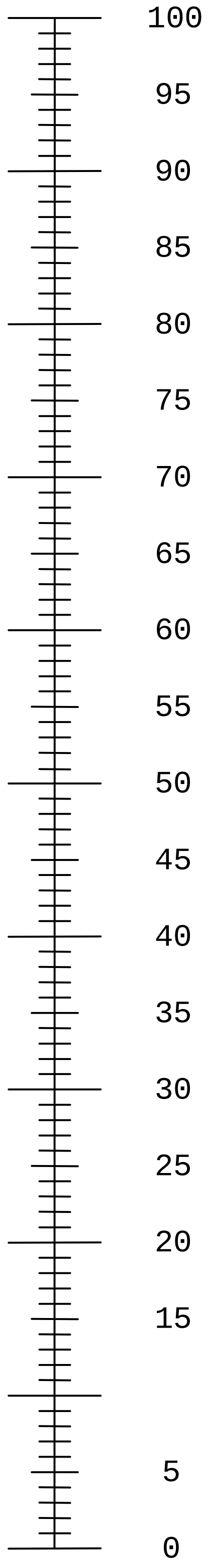

Schlechteste Gesundheit, die Sie sich vorstellen

15. Wir wollen herausfinden, wie gut oder schlecht Ihre Gesundheit HEUTE ist.

- Diese Skala ist mit Zahlen von 0 bis 100 versehen.
- 100 ist die <u>beste</u> Gesundheit, die Sie sich vorstellen können.
  0 (Null) ist die <u>schlechteste</u> Gesundheit, die Sie sich vorstellen können.
- Bitte kreuzen Sie den Punkt auf der Skala an, der Ihre Gesundheit HEUTE am besten beschreibt.
- Jetzt tragen Sie bitte die Zahl, die Sie auf der Skala angekreuzt
- haben, in das Kästchen unten ein.

IHRE GESUNDHEIT HEUTE = [ ]

VIELEN DANK

Germany (German) v.2 © 2010 EuroQol Group. EQ-5D™ is a trade mark of the EuroQol Group
